# Photosynthesis and growth reduction with warming are driven by nonstomatal limitations in a Mediterranean semi‐arid shrub

**DOI:** 10.1002/ece3.2074

**Published:** 2016-03-17

**Authors:** Lupe León‐Sánchez, Emilio Nicolás, Pedro A. Nortes, Fernando T. Maestre, José I. Querejeta

**Affiliations:** ^1^ Centro de Edafología y Biología Aplicada del Segura (CEBAS‐CSIC)Campus Universitario de EspinardoPO Box 164E‐30100MurciaSpain; ^2^ Biodiversity and Conservation UnitDepartment of Biology and GeologyUniversidad Rey Juan CarlosMóstoles28933MadridSpain

**Keywords:** Climate change, dryland ecosystems, *Helianthemum squamatum*, leaf trait plasticity, plant nutrient status, plant survival and growth, plant–climate interactions, stable isotopes

## Abstract

Whereas warming enhances plant nutrient status and photosynthesis in most terrestrial ecosystems, dryland vegetation is vulnerable to the likely increases in evapotranspiration and reductions in soil moisture caused by elevated temperatures. Any warming‐induced declines in plant primary production and cover in drylands would increase erosion, land degradation, and desertification. We conducted a four‐year manipulative experiment in a semi‐arid Mediterranean ecosystem to evaluate the impacts of a ~2°C warming on the photosynthesis, transpiration, leaf nutrient status, chlorophyll content, isotopic composition, biomass growth, and postsummer survival of the native shrub *Helianthemum squamatum*. We predicted that warmed plants would show reduced photosynthetic activity and growth, primarily due to the greater stomatal limitation imposed by faster and more severe soil drying under warming. On average, warming reduced net photosynthetic rates by 36% across the study period. Despite this strong response, warming did not affect stomatal conductance and transpiration. The reduction of peak photosynthetic rates with warming was more pronounced in a drought year than in years with near‐average rainfall (75% and 25–40% reductions relative to controls, respectively), with no indications of photosynthetic acclimation to warming through time. Warmed plants had lower leaf N and P contents, *δ* ^13^C, and sparser and smaller leaves than control plants. Warming reduced shoot dry mass production by 31%. However, warmed plants were able to cope with large reductions in net photosynthesis, leaf area, and shoot biomass production without changes in postsummer survival rates. Our findings highlight the key role of nonstomatal factors (biochemical and/or nutritional) in reducing net carbon assimilation rates and growth under warming, which has important implications for projections of plant carbon balance under the warmer and drier climatic scenario predicted for drylands worldwide. Projected climate warming over the coming decades could reduce net primary production by about one‐third in semi‐arid gypsum shrublands dominated by *H. squamatum*.

## Introduction

Anthropogenic greenhouse gas emissions are expected to raise global mean temperature by 2–6°C by the end of the XXI century (Collins et al. 2013). Manipulative field experiments have shown that warming generally enhances plant photosynthetic activity, primary production, and reproductive output in most ecosystem types (Rustad et al. 2001; Lin et al. 2010; Wu et al. 2011; Liang et al. 2013; Peñuelas et al. 2013). The positive effects of warming on primary production have been largely attributed to the kinetic sensitivity of photosynthesis to temperature and to extended growing seasons and enhanced nutrient availability (Luo 2007). Moreover, plants tend to increase their photosynthetic thermal optimum under warming, so photosynthesis acclimation to warming can further contribute to increase primary production when other resources, such as nutrients and water, are not limiting (Gunderson et al. 2010; Peñuelas et al. 2013).

Several lines of evidence indicate that plants may benefit more from warming in humid climates than in drier habitats (Rustad et al. 2001; Peñuelas et al. 2013; Xia et al. 2014; Tan et al. 2015). However, the generality of this rule has been questioned by both manipulative field studies and meta‐analyses (Perfors et al. 2003; Dijkstra et al. 2010, 2012; Lin et al. 2010; Wu et al. 2011). Compared with the large number of field warming studies conducted in tundra, alpine, boreal, and mesic temperate ecosystems, the impacts of warming on the nutrient status and photosynthesis of dryland vegetation have received less attention (but see Niu et al. 2008a,b; Dijkstra et al. 2010, 2012), even though dryland vegetation is particularly vulnerable to climate change (Peñuelas et al. 2013; Sardans and Peñuelas 2013). Arid, semi‐arid, and dry subhumid ecosystems (drylands hereafter) occupy 41% of the global land area (Safriel and Adeel 2005) and include about 20% of the major centers of global plant biodiversity (White and Nackoney 2003). Additional studies are thus urgently needed to improve our current understanding of the net impacts of warming on dryland plants, as any significant warming‐induced declines in primary production and vegetation cover in drylands would greatly increase erosion, land degradation, and subsequent desertification (Safriel and Adeel 2005; Wang et al. 2012).

The Mediterranean Basin will be particularly affected by climate change, as current general circulation models predict a 2–5°C warming during the coming decades, as well as longer, hotter, and drier summers with sharp reductions in late spring rainfall (up to 40%) and higher frequency and intensity of extreme droughts and heat waves (Giorgi and Lionello 2008; Collins et al. 2013). It is thus widely assumed that the net impacts of climate warming on the primary production of Mediterranean vegetation will be primarily dominated by increases in stomatal limitations on photosynthesis (Sardans and Peñuelas 2013; Bussotti et al. 2014; Nardini et al. 2014). In contrast to more northern latitudes, temperatures during the growing season in large parts of the Mediterranean Region may already be at or near the thermal optimum for plant photosynthesis (20–30°C for most species; Larcher 2000). Climate warming could enhance photorespiratory reactions (von Caemmerer and Quick 2000), impair the functioning of the photosynthetic biochemistry (Galmés et al. 2013), or even damage the leaf photosynthetic apparatus of many native plant species, particularly during summer drought periods when evaporative leaf cooling cannot prevent excessive leaf overheating (in which case leaf temperatures can reach up to 8–10°C higher than ambient temperatures; Larcher 2000).

Previous studies conducted in subhumid Mediterranean ecosystems have reported largely neutral or even positive effects of moderate nighttime warming (1°C) on the photosynthesis, nutrient status, and growth of native shrubs (Llorens et al. 2003; Peñuelas et al. 2004, 2007; Sardans et al. 2008a,b; Prieto et al. 2009a,b). However, the conclusions obtained from nighttime warming studies conducted in subhumid ecosystems cannot be extrapolated to semi‐arid environments, where photosynthesis is more severely water‐limited, and where vegetation could thus be more vulnerable to the desiccating effects of climate warming (Niu et al. 2008b). Moreover, plants can respond very differently to daytime versus nighttime warming (i.e., to increases in maximum vs. minimum daily temperatures), as increases in maximum daily temperatures can impose a greater constraint on both stomatal conductance and photosynthesis, and can thus be considerably more stressful for plants (Peng et al. 2013; Tan et al. 2015).

We conducted a four‐year manipulative field study in a semi‐arid Mediterranean ecosystem in southeast Spain to evaluate the effects of a 2°C warming on the leaf gas exchange, foliar N and P concentrations and isotopic composition (*δ* ^13^C, *δ* ^18^O), chorophyll content, leaf dry mass and area, shoot biomass production, and postsummer survival of the native shrub *Helianthemum squamatum* (L.) Dum. Cours. As water is by far the most limiting factor for primary productivity in semi‐arid Mediterranean ecosystems, we predicted that warmed plants would show reduced photosynthetic activity and growth, primarily due to the greater stomatal limitation imposed by faster and more severe soil drying and higher evaporative demand under warming (Maestre et al. 2013). Furthermore, we predicted that plant nutrient status would also be negatively affected by warming in this semi‐arid ecosystem, as the soil‐drying effects of warming can hamper organic N and P mineralization (Allison and Treseder 2008; Sardans and Peñuelas 2013) and decrease the geochemical desorption and dissolution of inorganic P (Dijkstra et al. 2012). The soil‐drying effects of warming would also be expected to reduce transpiration and thus the diffusion of nutrients to roots and the transport of nutrients from roots to leaves via the transpiration stream (He and Dijkstra 2014), a response with potential negative feedbacks on plant photosynthetic capacity (Wright et al. 2004).

## Material and Methods

### Study site and experimental design

This study was carried out near the town of Sax, in southeast Spain (38° 32′ 42″ N–0° 50′ 42″ W; 474 m.a.s.l.). The soil is derived from gypsum, has pH values ~7, and is classified as Gypsiric Leptosols (IUSS Working Group WRB, 2006). The vegetation is dominated by *Pinus halepensis*, which was planted in the 1950′s, and also contains grasses and shrubs, such as *Stipa tenacissima*,* Anthyllis cytisoides*, and *H. squamatum*. The climate is semi‐arid Mediterranean, with a mean annual temperature of 15°C and mean annual rainfall of 359 mm (28 years average; Villena weather station; 38° 37′ 46″ N–0° 51′ 40″ W; 486 m.a.s.l.).

We established a randomized field experiment to evaluate the effects of warming on the performance of *H. squamatum* shrubs. A passive warming treatment was achieved by installing open top chambers (OTCs) on vegetated patches dominated by *H. squamatum*. The OTCs have a hexagonal design with sloping sides of 40 cm × 50 cm × 32 cm and were made of transparent methacrylate. The methacrylate sheets used in our experiment transmit about 92% of visible light and over 85% of incoming energy and have a reflection of incoming solar radiation of 4% (according to the manufacturer; Decorplax S. L., Humanes, Spain). The methacrylate sheets used filtered up to 15% of UV radiation (Maestre et al. 2013). These OTCs have been used in previous field warming experiments (Maestre et al. 2013, 2015; Ladrón de Guevara et al. 2014). Upon installation in the field, the OTCs were suspended ~3 cm above the ground level by a metal frame to allow free air circulation and exchange with the surrounding environment (Fig. S1), which minimizes undesirable experimental effects, such as reduced wind and unnatural gas and humidity concentrations (Hollister and Webber 2000). The 2°C increase in mean annual air temperature achieved within the OTCs at our experimental site simulates warming levels within the range of predictions given by atmosphere–ocean general circulation models for the second half of the 21st century in southeastern Spain (Castro et al. 2005; Giorgi and Lionello 2008; Collins et al. 2013). Moreover, the OTCs used promote more intense warming effects during the summer than during winter (Maestre et al. 2013), which is also in good agreement with climate change model predictions for the region.

In October 2011, we installed nine OTCs in areas dominated by *H. squamatum* individuals. The same number of control plots (i.e., ambient temperature) was randomly established in adjacent areas containing *H. squamatum* individuals of similar size. To increase the number of replicates of each treatment, we also monitored *H. squamatum* individuals existing within OTCs that were established in 2008 in the framework of another study (Maestre et al. 2015) and that had exactly the same design and dimensions as those we installed. This increased the number of replicated control and warmed plots used in our study to 15, resulting in a total of 30 experimental plots. The leaf gas exchange rates of warmed *H. squamatum* shrubs did not differ significantly between OTCs established in 2008 versus OTCs established in 2011 (see Table S1).

To measure the effects of the OTCs on microclimatic parameters, we continuously monitored air temperatures, relative humidity, soil temperature, and soil moisture content (0–5 cm) in both warmed and control plots using replicated automated sensors (HOBO^®^ U23 Pro v2 Temp/RH sensors; Onset Corp., Pocasset, MA, USA, and EC‐5 soil moisture sensors; Decagon Devices Inc., Pullman, WA, USA, respectively).

### Plant measurements

Net photosynthesis rate (*A*), stomatal conductance (*g*_s_), transpiration rate (*E*), maximum efficiency of photosystem II under light (Fv'/Fm'), and the actual photochemical efficiency of photosystem II (∅PSII) were measured multiple times during 2012 (February, March, April, June, and November), 2013 (February, April, June, and November), 2014 (April), and 2015 (April) with a LI‐6400XT photosynthesis system (Li‐Cor, Inc., Lincoln, NE, USA) equipped with a LI‐6400‐40 Leaf Chamber Fluorometer and a LICOR 6400‐01 CO_2_ injector. Leaf gas exchange was measured on fully sun‐exposed leaves that were placed in a 2‐cm^2^ leaf cuvette. During these measurements, air CO_2_ concentration was controlled using the injection system and compressed CO_2_ cylinders with a CO_2_ concentration of 390 *μ*mol mol^−1^ CO_2_. Measurements were taken at a saturating light of 1500 *μ*mol m^−2^ s^−1^ and at ambient air temperature and relative humidity. The air flow was set to 250 *μ*mol s^−1^. All leaf gas exchange measurements were conducted between 8:00 and 11:00 am (GMT), when the peak of maximum photosynthetic rates was found at each survey. For warmed plants, all leaf gas exchange measurements were conducted under the prevailing microclimatic conditions within the OTCs (i.e., elevated temperature relative to ambient). All the leaves used for gas exchange measurements were collected thereafter to measure their area using an image scanner program (Image Pro Plus, Media Cybernetics, Inc. Rockville, MD, USA). On each date, leaf gas exchange measurements (*A*,* g*_s_, *E*, Fv'/Fm', and ∅PSII) were conducted on 10–15 *H. squamatum* individuals per each temperature treatment. Intrinsic water‐use efficiency (WUE_i_) and instantaneous water‐use efficiency (WUE_Inst_) were determined as *A*/*g*_s_ and *A*/*E*, respectively. Additionally, leaf respiration rates in the dark were measured in nine control and 12 warmed *H. squamatum* individuals at the peak of the 2014 growing season (April) to elucidate whether the large reduction in net photosynthetic rates found in warmed plants might be caused by increased leaf respiration rates. For calculations of mitochondrial respiration (nonphotorespiratory CO_2_ release), leaves were placed in the dark by covering them with aluminum foil for 30 min before CO_2_ exchange measurements, which were conducted at the same CO_2_ concentration as above, and with the air flow set to 150 *μ*mol s^−1^.

The leaves used for gas exchange measurements at spring (April) in each study year were collected thereafter to measure their carbon and oxygen isotope composition ratios (*δ* ^13^C and *δ* ^18^O, respectively). Samples were dried at 60°C and finely ground with a ball mill before being weighed and placed into tin capsules for these analyses. The *δ* ^13^C values and C and N concentrations of leaf material were measured by elemental analyzer/continuous flow isotope ratio mass spectrometry (ANCA/SL elemental analyzer coupled with a Finnigan MAT Delta PlusXL IRMS). The *δ* ^18^O of leaf material was determined with a Finnigan MAT Delta Plus XL IRMS (Finnigan MAT, Bremen, Germany) following the method described in Farquhar et al. (1997). Isotope analyses were conducted at the Centre for Stable Isotope Biogeochemistry, University of California, Berkeley (USA). Isotope ratios are expressed in delta notation (‰) relative to an internationally accepted reference standard: V‐PDB for *δ* ^13^C and V‐SMOW for *δ* ^18^O. Long‐term (3+ years) external precisions for *δ* ^13^C and *δ* ^18^O measurements of leaf material are 0.14 and 0.23‰, respectively. Leaf P concentrations were measured by inductively coupled plasma optical emission spectrometry (ICP‐OES; Thermo Elemental Iris Intrepid II XDL; Franklin, MA, USA) after a microwave‐assisted digestion with HNO_3_:H_2_O_2_ (4:1, v:v) in the Ionomics laboratory at CEBAS‐CSIC (Spain).

In April 2013, 2014, and 2015, we collected four leaves per plant (one individual per plot) to measure their dry mass, area, and leaf dry mass per unit area (LMA). Foliar areas were calculated using the Image Pro Plus software, and the leaves were thereafter oven‐dried at 60°C to determine their dry weight. LMA was calculated as the ratio between leaf dry weight and leaf area. In April 2015, we collected leaf material for chlorophyll content determinations. Briefly, 30 mg of leaves was sampled avoiding major veins, and chlorophyll was extracted from the leaves by submerging them in 3 mL of N,N‐dimethylformamide in the dark for at least 72 h. Absorbance was read at 647 nm and 664.5 nm with a Thermo Spectronic device (Helios alpha, UVA No. 092009, England) and used to calculate fresh mass‐based chlorophyll content (mg · g fresh mass^−1^) according to Inskeep and Bloom (1985).

Four years into the study (September 2015), we collected one representative terminal shoot per target plant (10 cm long, 15 plants per treatment) to evaluate the long‐term impacts of warming on the leaf and stem biomass production of *H. squamatum*. We measured total dry biomass (leaves plus stems) per unit shoot length, as well as the number of leaves and total leaf area per unit shoot length. More extensive destructive harvest of aboveground or belowground plant biomass (e.g. whole‐plant harvest) was ruled out to ensure the integrity of the target plants in this long‐term field experiment. Finally, we evaluated postsummer plant mortality after the first autumn rainfalls in each study year (October 2012–2015). Plant survival was measured as the percentage of surviving *H. squamatum* individuals in each experimental plot after the summer drought.

### Statistical analyses

We used repeated‐measures analysis of variance (RM‐ANOVA) to evaluate the effects of experimental warming on leaf gas exchange (*A*,* g*_s_, *E*, Fv':Fm', ∅PSII, WUE_i_, and WUE_Inst_; 10–11 sampling dates) and the variables that were measured at the peak of the growing season in each study year (leaf N and P concentrations, *δ* ^13^C, *δ* ^18^O, leaf dry mass and area, LMA, and postsummer plant survival). Temperature treatment (warming vs. control) and time were used as between‐subject and within‐subject factors in these analyses, respectively. Student's *t*‐tests were used to evaluate the effects of warming on shoot biomass production and leaf number and area. The relationships of leaf nutrient status with net photosynthetic rates and leaf biomass production were examined using linear regression analyses. To compare the intercepts and slopes of the regressions between *A* and *g*_s_ in the warming and control treatments, we used analysis of covariance (ANCOVA). RM‐ANOVA and ANCOVA analyses were performed using the software SPSS 19.0 (SPSS Inc., Chicago, IL, USA) and StatgraphicsPlus 5.1 (Statgraphics Plus 5.1. for Windows, 2000), respectively.

## Results

### Treatment effects on microclimatic variables and surface soil moisture content

Throughout the study period, the warming treatment (OTC) increased mean annual air temperature by ~2°C relative to ambient conditions (Fig. S2). Whereas minimum daily air temperatures were not significantly affected by the warming treatment, maximum daily air temperatures were on average 2.9°, 3.7°, 5.7°, and 3.7°C higher within the OTCs during winter, spring, summer, and autumn, respectively. Mean annual vapor pressure deficit was moderately but significantly higher in warmed plots (854 Pa) than in control plots (775 Pa), and this was attributable to differences in temperatures rather than to differences in mean annual relative humidity (which was similar in both treatments: 68.2% in control plots vs. 68.7% in warmed plots). Mean annual surface soil temperature (0–5 cm) was also higher in warmed plots (19.2°C) than in control plots (18.1°C). Mean daily surface soil temperatures were higher in warmed than in control plots by 0.7° in winter, by 1.3° in spring, by 2.1° in summer, and by 1.1 in autumn. The warming treatment increased maximum daily soil temperatures by 0.9° in winter, by 2.4° in spring, by 5.0° in summer, and by 1.2°C in autumn. Volumetric soil water content in the upper soil layer (0–5 cm depth) was lower in the warming treatment (8.0%) than in the control treatment (9.7%) across the study period (Fig. S3). Lower surface soil moisture content in the warming treatment was largely due to faster soil drying during rainless periods, rather than to any alteration of rainfall water inputs by the OTCs (as soil water content did not differ between warmed and control plots immediately after rain events).

### Rainfall variability during the study period and its relationship to the performance of *Helianthemum squamatum*

**Figure 1:**
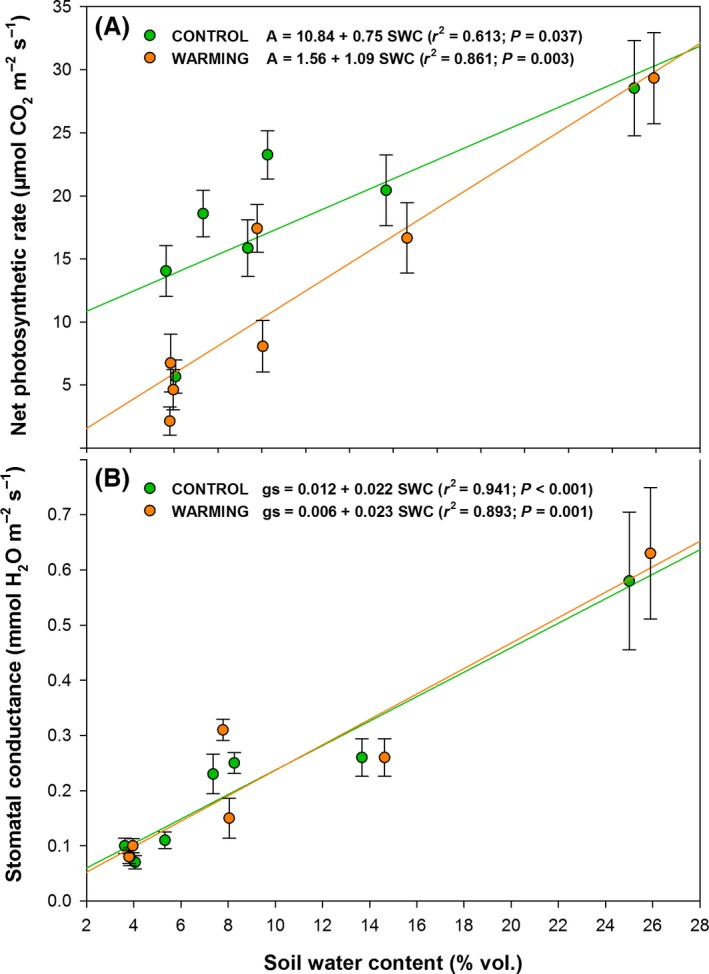
Linear regressions between net photosynthesis rate (A)/stomatal conductance (B) and soil water content in control and warmed plants across measurement dates. Each point represents the mean value of 10–15 replicated plants from separate plots. Vertical and horizontal error bars represent standard errors. Linear regression equations for control and warmed plants are also shown.

Rainfall varied dramatically during the study period (Fig. S3). Mean annual rainfall from October 2011 to September 2012 (hydrological year) was 307.2 mm (15% below average), whereas annual rainfall in the same period in 2012–2013 was 405 mm (13% above average). The 2013–2014 hydrological year was extremely dry, as total annual rainfall (141 mm) was 60% below average. The 2014–2015 hydrological year was near the average (378.6 mm). The study period included an unusually wet autumn (threefold higher rainfall than average in November 2012), a wet growing season (nearly twofold higher rainfall than average between February and April 2013), an exceptionally dry growing season (~27% of average rainfall during February–April 2014), as well as the second hottest summer in the last 60 years (24.1°C mean temperature in Summer 2015). Across temperature treatments, stomatal conductance and net photosynthetic rates were strongly limited by soil water availability (Fig. 1). Moreover, all the variables measured in *H. squamatum* at the peak of the growing season were strongly affected by the high interannual climatic rainfall variability during the study period (Table S2). Across temperature treatments, net photosynthetic rates were highest during wet periods in early spring when ambient air temperature at midday was between 17–27°C, and photosynthesis decreased sharply above 30°C.

### Warming impacts on leaf gas exchange

**Figure 2:**
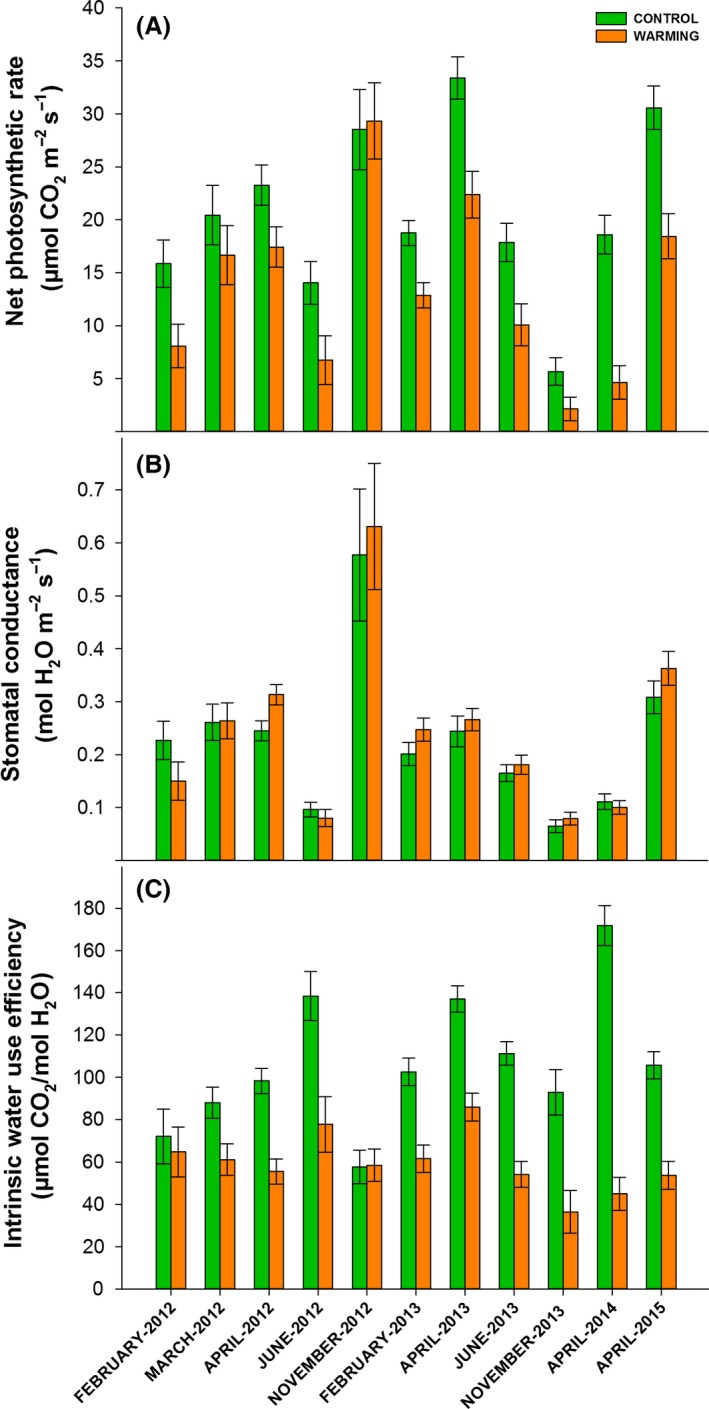
Mean net photosynthetic rates (A), stomatal conductance (B), and intrinsic water‐use efficiency values (C) in warmed and control plants at 11 different measurement dates spanning four growing seasons. Data represent means ± SE (*n* = 10–15).

**Figure 3:**
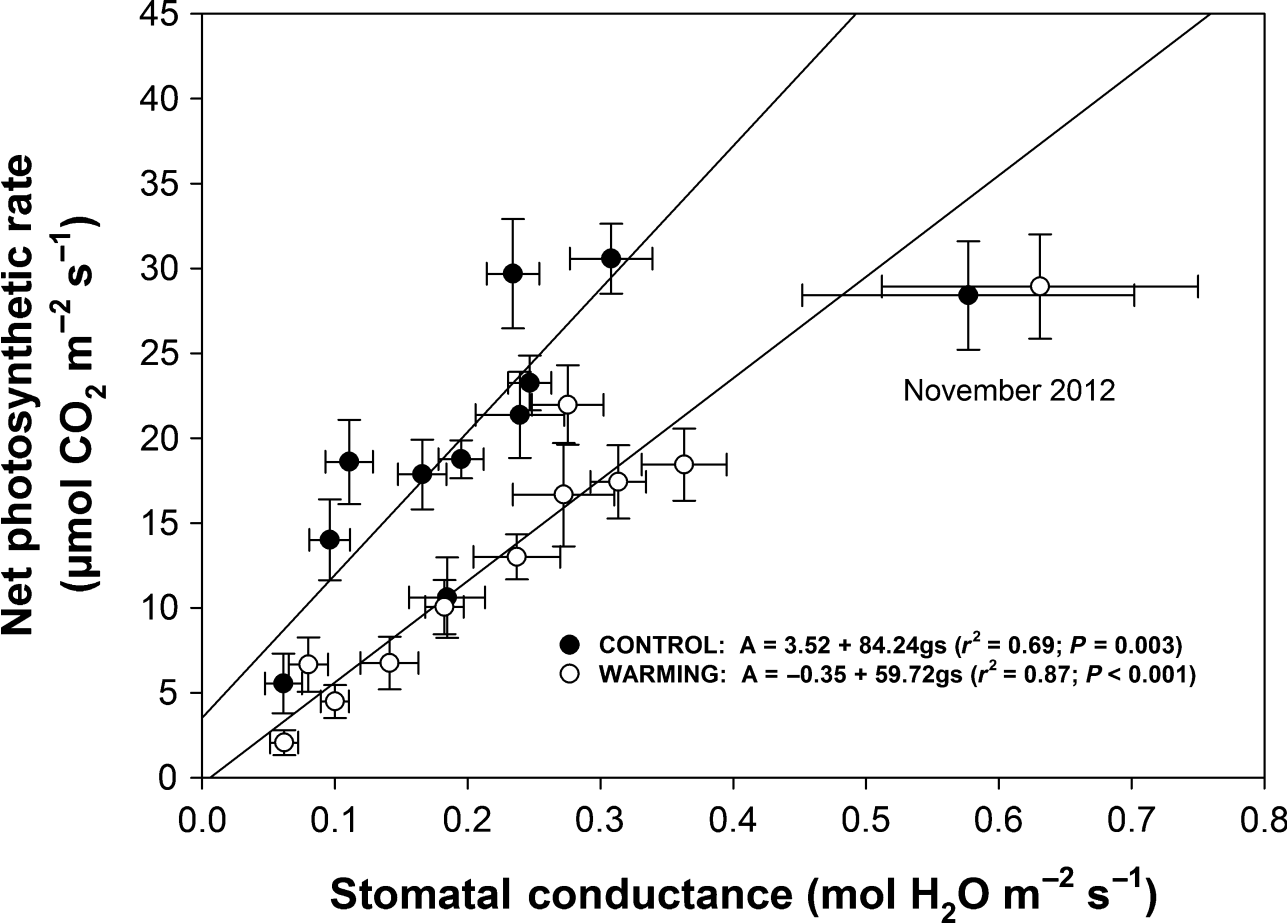
Linear regressions between mean net photosynthesis rates (*A*) and mean stomatal conductance (*g*_s_) in control and warmed plants across measurement dates. Each point represents the mean value of 10–15 replicated plants from separate plots. Vertical and horizontal error bars represent standard errors. Fitted linear regressions for control and warmed plants are also shown. Analysis of covariance indicated that the regression lines of control and warmed plants were significantly different (*P* = 0.001). Data recorded in November 2012 were excluded from the regression analysis, as there were no significant differences in *A* or *g*_s_ between temperature treatments at this time.

**Figure 4:**
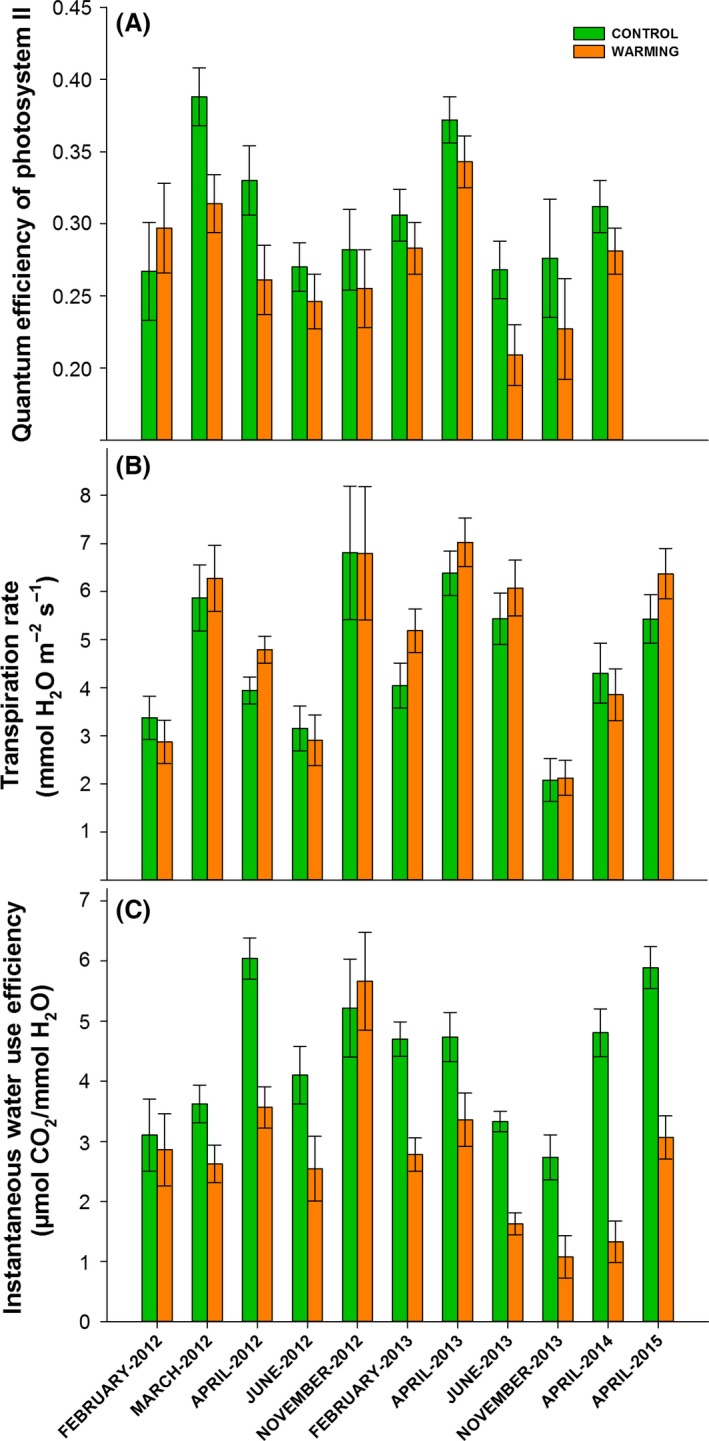
Quantum efficiency of photosystem II (A), transpiration rates (B), and instantaneous water‐use efficiency values (C) in warmed and control plants at 10–11 different measurement dates spanning four consecutive growing seasons. Data represent means ± SE (*n* = 10–15).

The warming treatment consistently reduced the net photosynthetic rates (*A*), intrinsic water‐use efficiency (WUEi), and instantaneous water‐use efficiency (WUE_Inst_) of *H. squamatum* throughout the study period (*P* < 0.001 in all cases, Table S2; Figs. 2A, C and 4C, respectively). Averaged across growing seasons and measurement dates, mean *A* and WUEi values were ~36% and 41% lower, respectively, in warmed than in control plants. The strong detrimental effects of warming on *A* and WUE were exacerbated during drought periods, as indicated by their respective significant *Warming × Year* interactions (Table S2). The reduction of peak photosynthetic rates during spring in warmed plants was more pronounced in a severe drought year (75% reduction relative to controls in April 2014) than in years with near‐average rainfall (25%, 35%, and 40% reductions in April 2012, 2013, and 2015, respectively). Interestingly, the only time when *A* and WUE values did not differ between warmed and control plants was during a cool and unusually rainy period in autumn (November 2012, with 15.7°C mean monthly temperature, 16 rainy days and threefold higher rainfall than average; Fig. 2A, C). In contrast to the large and consistent differences in *A* and WUEi between warmed and control plants, stomatal conductance (*g*_s_) and transpiration rate (*E*) did not vary significantly with warming (*P* = 0.400 and *P* = 0.690, respectively, Table S2; Figs. 2B and 4B, respectively). When pooling leaf gas exchange data across measurement dates, the linear regression between net photosynthetic rate and stomatal conductance had lower intercept and slope values for warmed plants than for control plants (Fig. 3).

The maximum efficiency of photosystem II (Fv':Fm') was not affected by the warming treatment (*P* = 0.870, Table S2; Fig. S4). However, the warming treatment marginally reduced the quantum efficiency of photosystem II (∅PSII) in *H. squamatum*, although this effect was much smaller and less consistent through time than the effects on *A* and WUEi (*P* = 0.058, Table S2; Fig. 4A). Across study years, ∅PSII values at the peak of the growing season (April) were on average 21% lower in warmed plants than in control plants. Leaf respiration rates in the dark (mitochondrial respiration) did not differ between warmed and control plants at the peak of the growing season in 2014 (means ± standard errors = −1.43 ± 0.33 vs. −1.33 ± 0.38 *μ*mol CO_2_ m^2^ s^−1^, respectively; *F* = 0.033; *P* = 0.858; *n* = 21), although this result must be interpreted with caution due to the limited scope of the measurements.

### Warming impacts on leaf nutrients, *δ* ^13^C, *δ* ^18^O, chlorophyll contents, leaf dry mass, and area

The warming treatment consistently reduced leaf N and P concentrations at the peak of the growing season (*P* = 0.024 and *P* = 0.005, respectively, Table S3; Table 1). Across study years, mean leaf N and P were on average 5.2% and 12.7% lower in warmed plants than in control plants, respectively. As a result, mean leaf C/N and C/P ratios were up to 11% and 16% higher, respectively, in warmed than in control plants. However, during a cool and unusually rainy period in autumn 2012, foliar N and P concentrations did not differ between control and warmed plants (*N* = 2.1 ± 0.1% in both treatments; *P* = 1.3 ± 0.1 and 1.4 ± 0.1 mg g^−1^ in control and warmed plants, respectively). Across study years, peak photosynthetic rates during spring correlated positively with leaf N and P concentrations in warmed plants (*r* = 0.536, *P* < 0.001 and *r* = 0.294, *P* = 0.024, respectively; *N* = 60), suggesting nutritional limitation of photosynthesis under warming. In contrast, interannual variation in peak photosynthetic rates was unrelated to leaf N or P status for control plants (*r* = −0.017, *P* = 0.899 and *r* = 0.173, *P* = 0.187, respectively; *N* = 60).

**Table 1:** Leaf N and P concentrations, leaf dry mass, area, and leaf mass per unit area (LMA) values, leaf *δ* ^13^C and *δ* ^18^O, and postsummer plant survival values in the four years of the study. Data represent means ± SE (*n* = 10–15)

	Leaf N (%)	Leaf P (mg g^−1^)	Leaf mass (mg)	Leaf area (cm^2^)	LMA (mg/cm^2^)	Leaf *δ* ^18^O	Survival
2012							
Control	2.60 ± 0.07	1.27 ± 0.06	–	–	–	26.42 ± 0.35	63.6
Warming	2.22 ± 0.07	1.06 ± 0.06	–	–	–	26.16 ± 0.35	55.3
2013							
Control	2.20 ± 0.07	1.01 ± 0.05	12.12 ± 0.59	0.74 ± 0.04	16.5 ± 0.5	25.35 ± 0.24	94.7
Warming	2.15 ± 0.08	0.87 ± 0.06	10.28 ± 0.70	0.69 ± 0.05	14.6 ± 0.6	25.25 ± 0.27	100.0
2014							
Control	1.73 ± 0.06	0.91 ± 0.06	10.69 ± 0.714	0.60 ± 0.05	17.9 ± 0.5	25.60 ± 0.39	33.3
Warming	1.57 ± 0.06	0.79 ± 0.05	8.83 ± 0.66	0.55 ± 0.05	18.2 ± 0.5	26.98 ± 0.35	28.9
2015							
Control	2.44 ± 0.05	0.89 ± 0.04	13.73 ± 1.06	1.11 ± 0.08	12.5 ± 0.6	24.10 ± 0.22	94.4
Warming	2.34 ± 0.06	0.79 ± 0.05	12.58 ± 1.10	1.05 ± 0.08	11.9 ± 0.5	23.41 ± 0.21	89.2

**Figure 5:**
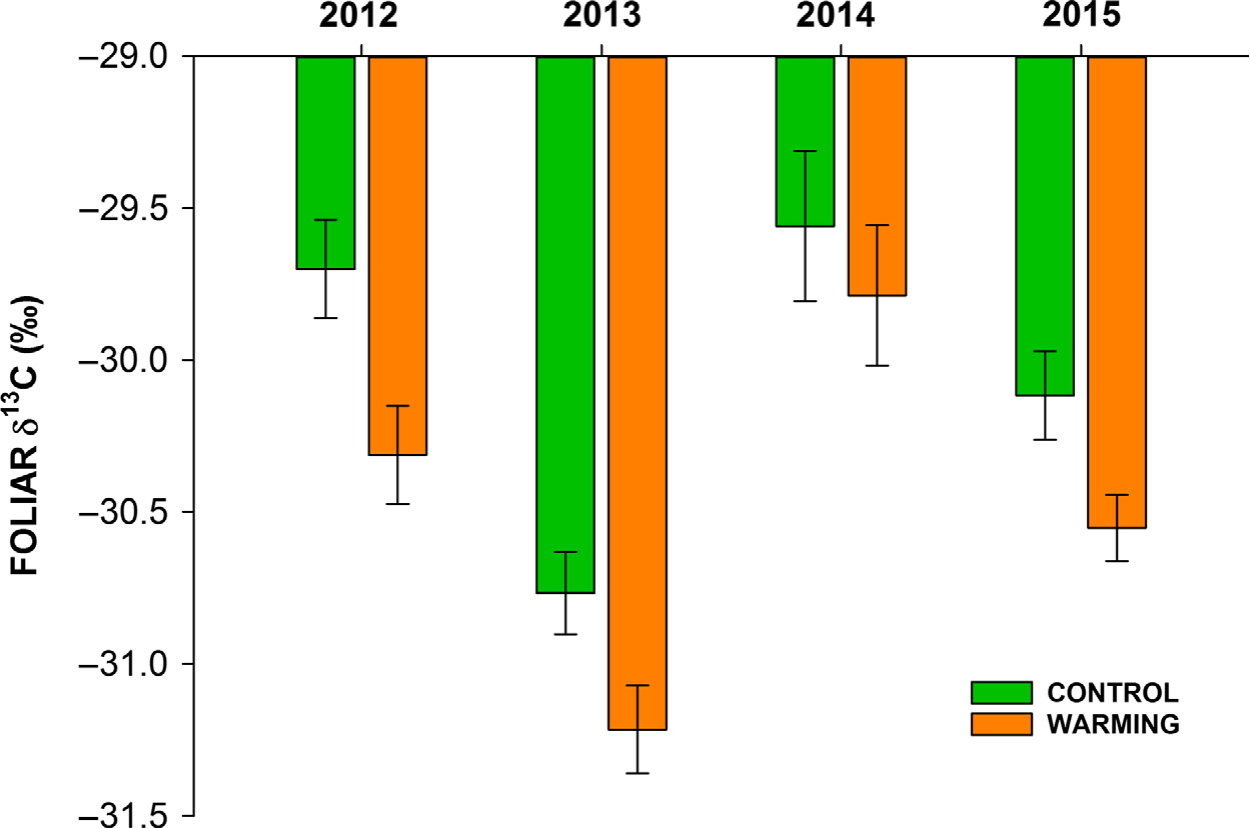
Mean leaf *δ* ^13^C values in control and warmed plants at the peak of the growing season (April) in the 4 years of the study. Data represent means ± SE (*n* = 10–15).

Across study years, warmed plants showed consistently lower leaf *δ* ^13^C values than control plants (*P* = 0.016, Table S3; Fig. 5). However, the leaf *δ* ^18^O values of *H. squamatum* shrubs were not consistently affected by the warming treatment across years (*P* = 0.735, Table S3; Table 1). Warmed plants showed a higher leaf chlorophyll b content than control plants (0.300 ± 0.007 and 0.275 ± 0.010 mg/g, respectively; *F* = 4.194; *P* = 0.055) in April 2015. Chlorophyll a content was not significantly affected by warming (0.979 ± 0.034 mg/g in control plants and 0.976 ± 0.026 mg/g in warmed plants; *F* = 0.006; *P* = 0.941). The chlorophyll a:b ratio was thus significantly lower in warmed plants than in plants exposed to ambient temperature conditions (3.275 ± 0.034 and 3.524 ± 0.047, respectively; *F* = 13.366; *P* = 0.001).

**Figure 6:**
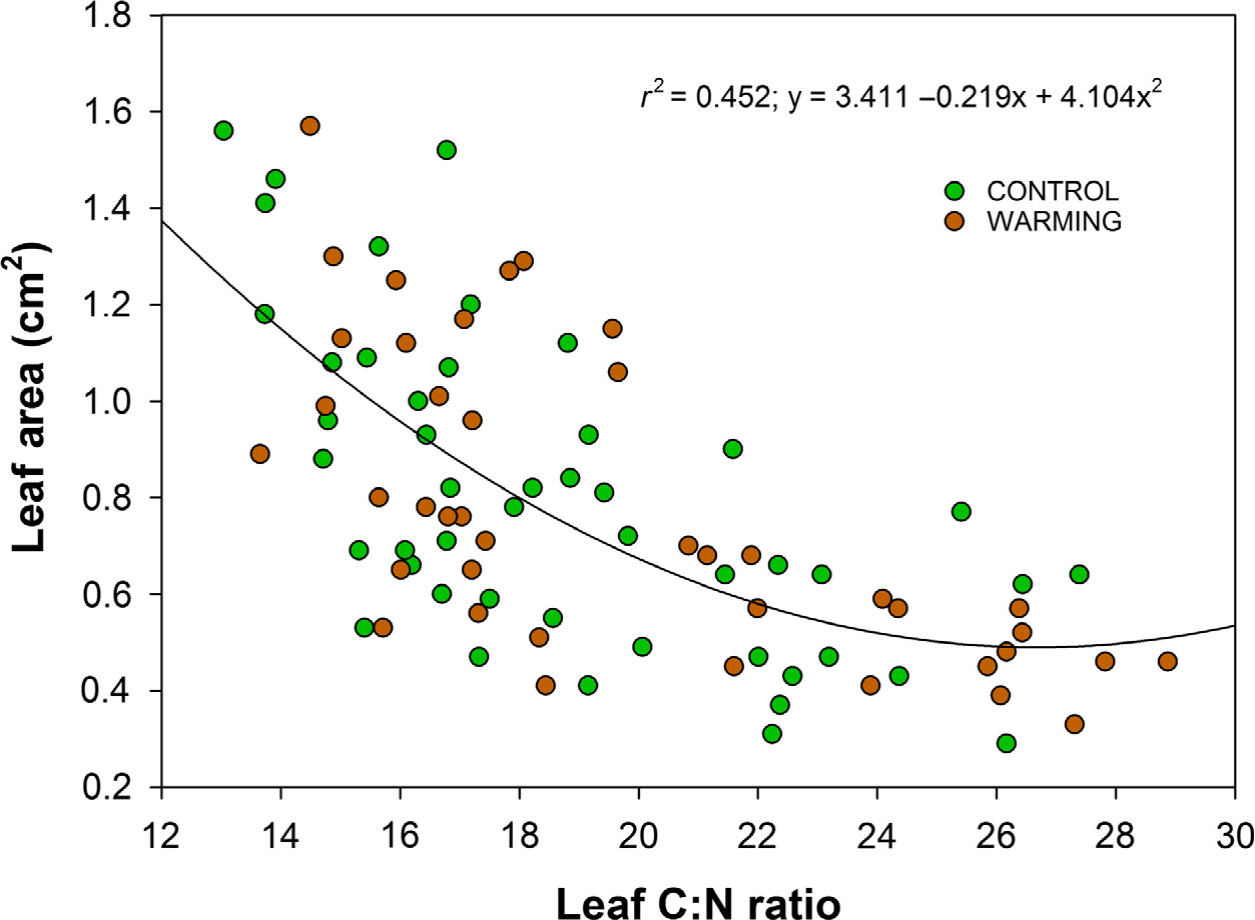
Negative relationship between leaf areas and C:N ratios at the peak of the growing season (April 2013, 2014, 2015). Green circles represent control plants and orange circles represent warmed plants. *N* = 10–15 individual plants per treatment × year combination.

Leaf dry mass at the peak of the growing season (April) was negatively correlated with leaf C:N (*r* = −0.445; *P* < 0.001; *N* = 89) and C:P (*r* = −0.263; *P* = 0.013; *N* = 89) ratios across years and temperature treatments, suggesting nutrient‐limited leaf growth. Leaf area at the peak of the growing season also correlated negatively with leaf C:N (*r* = −0.641; *P* < 0.001; *N* = 89; Fig. 6) and C:P (*r* = −0.246; *P* = 0.021; *N* = 89) ratios across years and treatments. Averaged across years, mean leaf dry mass and area were 13% and 11% lower, respectively, in warmed plants than in control plants (*P* = 0.044 and *P* = 0.093, respectively, Table S3; Table 1). The relative decreases in mean leaf dry mass and area under warming were greatest during the drought year (2014, 17.5% and 21% decreases, respectively). The simultaneous decreases in leaf dry biomass and leaf N and P concentrations under warming translated into large relative decreases in leaf N and P contents across years (18% and 25% decreases, respectively).

### Warming impacts on shoot biomass growth and plant postsummer survival

Four years after the start of the experiment, the total dry biomass of terminal shoots (foliage plus stems) per unit length was 31% lower in warmed plants than in control plants (Table 2). Warmed plants had on average 35% lower leaf dry mass, 19% lower stem dry mass, 23% lower number of leaves, and 29% smaller leaf area per unit shoot length than control plants (Table 2). However, postsummer plant survival rates did not differ between warmed and control plots within or across years (*P* = 0.832, Table S3; Table 1).

**Table 2:** Mean number of leaves, total leaf area, total dry mass (leaves plus stem), leaf dry mass, and stem dry mass of terminal shoots (10 cm long) in control and warmed *Helianthemum squamatum* plants. Data represent means ± SE (*n* = 15 plants per treatment). *P*‐values <0.05 indicate significant differences between temperature treatments (*t*‐test)

	Leaf number	Leaf area (cm^2^)	Shoot dry mass (mg)	Leaf dry mass (mg)	Stem dry mass (mg)
Control	81.7 ± 4.9	17.0 ± 1.4	360 ± 26	268 ± 22	91 ± 5
Warming	62.6 ± 4.8	12.0 ± 1.3	248 ± 25	173 ± 21	74 ± 4
*P*‐value	0.010	0.014	0.004	0.004	0.013

## Discussion

### Warming effects on leaf gas exchange, nutrient, and chlorophyll concentrations

Simulated climate warming caused large reductions of net photosynthetic rates in *H. squamatum*, particularly during dry periods, whereas stomatal conductance and transpiration rates remained largely unchanged. Therefore, decreased net photosynthetic rates in warmed plants were not caused by increased stomatal limitations on *A*, but rather by nonstomatal constraints (nutritional, biochemical, metabolic, and/or diffusional; Flexas and Medrano 2002; Galmés et al. 2007; Flexas et al. 2014). Warming reduced the carbon assimilation capacity of *H. squamatum* leaves at any given stomatal aperture (Fig. 3), except during a cool and unusually rainy period in November 2012. Furthermore, there was no evidence of photosynthesis acclimation to warming over the four years studied (Gunderson et al. 2010); on the contrary, the negative effects of warming on *A* and WUEi were most pronounced in the last two years of the study (Fig. 2A, C). In agreement with leaf gas exchange data, warmed plants showed consistently lower leaf *δ* ^13^C values than control plants across years (Fig. 5), which indicates that warming decreased time‐integrated water‐use efficiency through nonstomatal reductions in net photosynthesis (Scheidegger et al. 2000; Seibt et al. 2008; Cernusak et al. 2013). Moreover, leaf *δ* ^18^O was not consistently affected by warming across years, which further suggests that neither time‐integrated stomatal conductance (Fig. 2B), nor the time‐integrated *δ* ^18^O of the source water (which is primarily determined by rooting depth and soil water uptake depth) may have differed greatly between warmed and control plants (Barbour 2007).

Identifying and disentangling the underlying physiological mechanisms responsible for the large reduction of net photosynthetic rates under moderate warming in *H. squamatum* shrubs are beyond the scope of this study. Decreased photosynthetic rates with moderate temperature increases above the photosynthetic thermal optimum (see Galmés et al. 2013 for a physiologically based definition of “moderate” vs. “severe” plant heat stress) have been previously reported by many glasshouse studies (Sage and Kubien 2007; Galmés et al. 2013). Decreased photosynthesis under moderate warming has been related to biochemical limitations, including, among others: (a) enhanced photorespiration due to decreased Rubisco specificity for CO_2_ relative to its alternative substrate oxygen and increased solubility of oxygen relative to CO_2_ with rising temperature (von Caemmerer and Quick 2000); (b) heat‐induced inhibition of ribulose‐1.5‐bisphosphate regeneration (Kubien and Sage 2008); (c) downregulation of Rubisco activation state caused by increased catalytic inactivation of Rubisco at elevated temperatures and by the high thermal lability of the enzyme Rubisco activase (Crafts‐Brandner and Salvucci 2000; Sage and Kubien 2007); (d) decreases in leaf protein content and Rubisco concentration under warming (Galmés et al. 2013); and (e) reduced electron transport capacity of photosystem II at supraoptimal temperatures for photosynthesis (Sage and Kubien 2007).

Warmed plants exhibited large reductions in *A* and WUEi even at the peak of the spring growing season, which is a period of mild temperatures in our study area (Fig. S2) and considered optimal for photosynthesis (Galmés et al. 2013). The moderate increase in mean air temperature achieved by the warming treatment during spring (~1.5–2°C) is thus unlikely to have caused any severe or irreversible heat‐induced damage of the leaf photosynthetic machinery in warmed plants. Leaf gas exchange measurements were always conducted on young leaves formed during the current spring, so carryover heat stress damage from the previous summer (maximum daily temperatures during summer within the OTCs increased up to 6°C) is also an implausible explanation for the large reductions of *A* in warmed plants during the subsequent growing season. Interestingly, the maximum efficiency of photosystem II under light conditions (Fv':Fm'; Baker and Rosenqvist 2004) was not negatively affected by the warming treatment (Fig. S4), which does not support a damaged PSII phytochemistry in warmed plants. Therefore, marginally decreased ∅PSII in warmed plants probably reflects PSII downregulation in response to a reduced carboxylation capacity (Loik et al. 2000; Baker and Rosenqvist 2004; Sage and Kubien 2007).

The tight negative correlations found between leaf dry mass/area production and C:N and C:P ratios indicate nutrient limitation of foliar growth in *H. squamatum*. The simultaneous decreases of leaf N and P concentrations in warmed plants thus likely caused interactive and mutually reinforcing detrimental effects on their photosynthetic performance, given the key importance of N and P for Rubisco carboxylation capacity and for ribulose‐1.5‐bisphosphate regeneration, respectively (Warren and Adams 2002; Campbell and Sage 2006; Reich et al. 2009). Leaf photosynthetic capacity is strongly correlated with leaf N concentration because of the crucial role that N‐rich enzymes, particularly Rubisco, play in the biochemical fixation of CO_2_ (Field and Mooney 1986; Wright et al. 2004). Low leaf P concentration further limits photosynthesis through reductions in ribulose‐1,5‐bisphosphate regeneration, photochemical efficiency of photosystem II, mesophyll conductance to CO_2,_ and stomatal conductance (Jacob and Lawlor 1991, 1992; Loustau et al. 1999; Thomas et al. 2006). Interestingly, the single measurement date when *A* and WUE values did not differ between warmed and control plants (during a cool and unusually wet period in November 2012) coincided with the only time when leaf N and P concentrations did not differ between treatments either. This result supports a role of decreased foliar N and P status in reducing photosynthesis in warmed plants at all other measurement dates.

Faster and more severe drying of the fertile upper soil layers (0–5 cm) where plant‐available nutrients are most abundant may have hampered nutrient mineralization, solubilization, diffusion, and/or uptake by roots in the warmed plots (Sardans and Peñuelas 2013), which could explain the lower leaf N and P status of warmed plants relative to control plants. Decreased leaf N and P concentrations in warmed plants (along with lower leaf *A*, WUEi, *δ* ^13^C and biomass growth) would also be consistent with a decreased mycorrhizal contribution to nutrient uptake, as mycorrhizal fungi usually enhance the nutrient status, photosynthesis and water‐use efficiency (through C sink stimulation), *δ* ^13^C, and growth of their host plants in semi‐arid ecosystems (Querejeta et al. 2006; Mohan et al. 2014). Further research is warranted to resolve the precise mechanisms responsible for the moderate but consistent decreases in leaf N and P found under warming.

Contrary to expectations, warming did not alter the stomatal conductance and transpiration rates of *H. squamatum* (Table S2; Figs. 2B and 4B), despite faster and more severe surface soil drying (0–5 cm, Fig. S3) and higher evaporative demand in warmed plots, thus suggesting anisohydric behavior in this species (Tardieu and Simonneau 1998). *H. squamatum* shrubs likely have the ability to extract water from deeper (> 5 cm) soil layers that are less intensely affected by the drying effects of warming than surface layers. Mediterranean woody plants have evolved a wide array of adaptive homeostatic mechanisms to cope with fast and intense soil drying (e.g., adjustments in root: shoot biomass ratios, rooting depth or volume, and/or hydraulic architecture; Nardini et al. 2014), which may have enabled *H. squamatum* plants to sustain unchanged *g*_s_ and *E* under warming. In addition, the ability of *H. squamatum* shrubs to use the crystallization water of gypsum rocks and soils as a water source (Palacio et al. 2014) may have further helped them to maintain unchanged *g*_s_ and *E* under warming, given that the release of gypsum crystallization water is favoured by warming. Moreover, the 25% reduction in leaf area per unit shoot length in warmed plants resulted in sparser foliage, which may have allowed them to sustain unchanged *g*_s_ and *E* rates on a leaf area basis, while at the same time reducing total canopy transpiration (Limousin et al. 2009).

Leaf photosynthetic pigment ratios, such as the chlorophyll a: b ratio, provide useful indicators for plant stress detection, including heat, drought, and nutrient stresses and their combination (Thompson et al. 1992; Zhang et al. 2008). The chlorophyll a:b ratio decreased significantly in the warmed plants as compared with the controls, which is an indication of severe stress and is in accordance with the large reductions in net photosynthetic rates and shoot biomass production found under warming.

### Warming effects on plant growth and survival

Interestingly, warmed plants were able to achieve similar postsummer survival rates as control plants throughout the study, despite their lower leaf area and biomass, foliar nutrient contents, and photosynthetic rates. Given that the study period encompassed both the driest hydrological year on record (2013–2014) and the second hottest summer on record (2015) across southeastern Spain, the unchanged survival rates under warming highlight the remarkable resistance and resilience of *H. squamatum* shrubs against forecasted climate change conditions. However, plant growth was strongly reduced by moderate warming, as warmed plants had about one‐third lower shoot dry biomass production (leaves plus stems) and leaf area at the end of the study period. The lower carbon cost of building thinner stems and sparser and smaller leaves (Table 2) may be a key adaptive mechanism that helped maintain the carbon balance and survival of *H. squamatum* shrubs under warming, despite the large reductions in their net carbon assimilation rates (Nicotra et al. 2010; Bussotti et al. 2014). Reducing leaf numbers and leaf area per unit shoot length may also be an effective adaptive mechanism to decrease total canopy transpiration under warming, in order to minimize the risk of catastrophic hydraulic failure under enhanced vapor pressure deficit and soil drying (Limousin et al. 2009).

### Comparison with other warming experiments

The strong detrimental effects of simulated climate warming on photosynthesis and growth in this semi‐arid ecosystem contrast to the overwhelmingly positive or neutral plant responses to warming found in most terrestrial ecosystems (including drylands; Rustad et al. 2001; Xia et al. 2009; Raich et al. 2006; Lin et al. 2010; Wu et al. 2011; Peñuelas et al. 2013). Few (if any) field studies have so far reported such large reductions in peak photosynthesis rates (25–75%) and plant biomass growth (31%) linked to simultaneous decreases in leaf N and P concentrations because of warming. The vast majority of field studies conducted to date report increased leaf N and/or P concentrations (attributed to increased soil nutrient mineralization) and photosynthesis under warming (Rustad et al. 2001; Melillo et al. 2002; Butler et al. 2012; Dijkstra et al. 2012; Bai et al. 2013). Although a few studies found decreases in leaf N concentrations under warming, this was generally attributed to dilution effects caused by enhanced photosynthesis and growth (An et al. 2005; Dijkstra et al. 2010), or to changes in proportional nutrient allocation to leaves versus woody tissue in warmed plants (Sardans et al. 2008a).

In a series of field studies investigating the responses of native shrubland species to passive nighttime warming in a subhumid Mediterranean environment, Llorens et al. (2003) found few consistent changes in *A*, WUEi, foliar N, or leaf *δ* ^13^C values in warmed relative to control plants. However, the nutrient status and photosynthetic performance of some shrub species were moderately enhanced by warming, as indicated by increased foliar P, higher Rubisco activity and carboxylation efficiency, enhanced photochemical efficiency of photosystem II, and increased biomass production (Llorens et al. 2003; Sardans et al. 2008b; Prieto et al. 2009a,b). The discrepancies between our study and previous work thus demonstrate that climate warming can be much more detrimental to plant nutrient status, photosynthesis, and growth in semi‐arid Mediterranean ecosystems than previously reported for subhumid Mediterranean sites (Llorens et al. 2003; Peñuelas et al. 2004, 2007; Prieto et al. 2009a,b). Differences in the timing (daytime vs. nighttime) and intensity of warming (2°C vs. 1°C) could in part explain the contrasting results with these previous studies. However, the large differences in annual rainfall between study sites likely play a more pivotal role in determining plant vulnerability to warming (Swarbreck et al. 2011): whereas mean annual temperature was very similar between our semi‐arid site and the abovementioned subhumid site (around 15°C; Llorens et al. 2003), mean annual rainfall during the respective study periods was much lower at our site (308 vs. 580 mm; Llorens et al. 2003). In support of this interpretation, we found that the detrimental effects of warming on the *A*, WUE, and leaf N and P concentrations of *H. squamatum* shrubs disappeared transiently during an unusually rainy period in November 2012.

### Concluding remarks

A moderate (~2°C) warming exerted strong negative effects on the net photosynthetic rate and shoot biomass growth of *H. squamatum*, especially (but not only) during dry periods. Warming reduced peak photosynthetic rates during spring by 25–40% in near‐average rainfall years and by 75% in a severe drought year, which has important implications for projections of plant carbon balance under the warmer climatic scenario predicted for the Mediterranean (Giorgi and Lionello 2008) and other dryland regions. However, warmed plants were able to cope with large reductions in net photosynthetic rates, leaf area, and shoot biomass production without significant changes in postsummer plant survival. Contrary to expectations, stomatal conductance, and transpiration rates remained unchanged under warming, thus highlighting the key role of nonstomatal limitations on photosynthesis (e.g., biochemical and nutritional; Flexas et al. 2014) in this Mediterranean shrub. Our findings indicate that warming could significantly reduce net primary production (by about one‐third) and potentially alter other key ecological processes such as plant–herbivore relationships, leaf litter decomposition, and nutrient cycling (through changes in leaf N and P) in semi‐arid gypsum shrublands dominated by *H. squamatum*.

## Conflict of Interest

None declared.

## Supporting information


**Appendix S1.** Supporting information figures and tables.
