# Supplementary material for: Photosynthesis and growth reduction with warming are driven by nonstomatal limitations in a Mediterranean semi‐arid shrub
Source: Ecol Evol. 2016 Mar 17;6(9):2725–38. doi: 10.1002/ece3.2074 (PMC4798828; doi:10.1002/ece3.2074)
Supplement: Supplementary file 1 — Appendix S1. Supporting information figures and tables. [file ECE3-6-2725-s001.docx]

## Supporting Information

Article title: **Photosynthesis reduction with warming is driven by non-stomatal limitations in a Mediterranean semiarid shrub**

Authors: Lupe León-Sánchez, Emilio Nicolás, Pedro A. Nortes, Fernando T. Maestre, José I. Querejeta

The following Supporting Information is available for this article:

**Fig. S1.** Detailed view of an open top chamber (OTC) including several *H. squamatum* shrubs. **Fig. S2.** Mean air temperature in the control and warming treatments throughout the experiment.

**Fig. S3.** Rainfall distribution and soil water content in warmed and control plots throughout the experiment.

**Fig. S4.** Maximum efficiency of photosystem II under light conditions (Fv’:Fm’) in control and warmed plants.

**Table S1.** Mean net photosynthetic rate, stomatal conductance and intrinsic water use efficiency at 11 different measurement dates spanning 4 growing seasons in warmed plants of OTCs installed in 2008 versus warmed plants of OTCs installed in 2011.

**Table S2.** Results of the repeated measures analysis of variance (RM-ANOVA) of net photosynthetic rate (A), stomatal conductance (g_s_), transpiration rate (E), intrinsic water use efficiency (WUE_i_), instantaneous water use efficiency (WUE_Inst_), maximum efficiency of photosystem II under light conditions (Fv’:Fm’) and the actual photochemical efficiency of photosystem II (∅PSII).

**Table S3.** Results of the repeated measures analysis of variance (RM-ANOVA) of leaf N and P concentrations, leaf mass, area and mass per unit area (LMA, data not available for April 2012), leaf isotopic composition (δ^13^C, δ^18^O, measured in April 2012, 2013, 2014, 2015), and post-summer plant survival (measured in October 2012, 2013, 2014 and 2015).

**Fig. S1** Detailed view of an open top chamber (OTC), including several *H. squamatum* shrubs. The bottom of the OTC is elevated ~3 cm above the ground level to allow ventilation and avoid overheating.


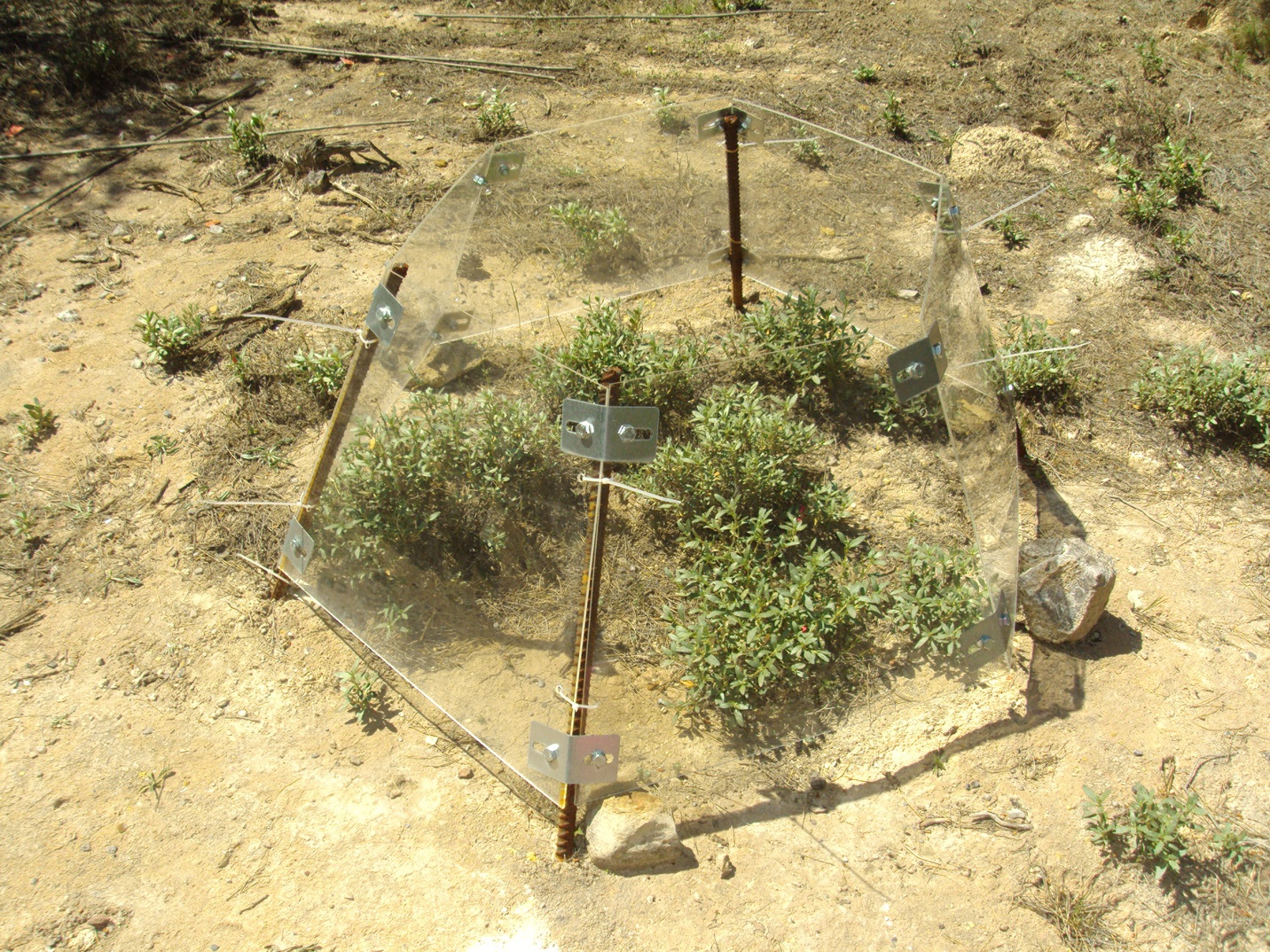


**Fig. S2** Air temperature in the control and warming treatment throughout the experiment, from October 2011 until October 2015.


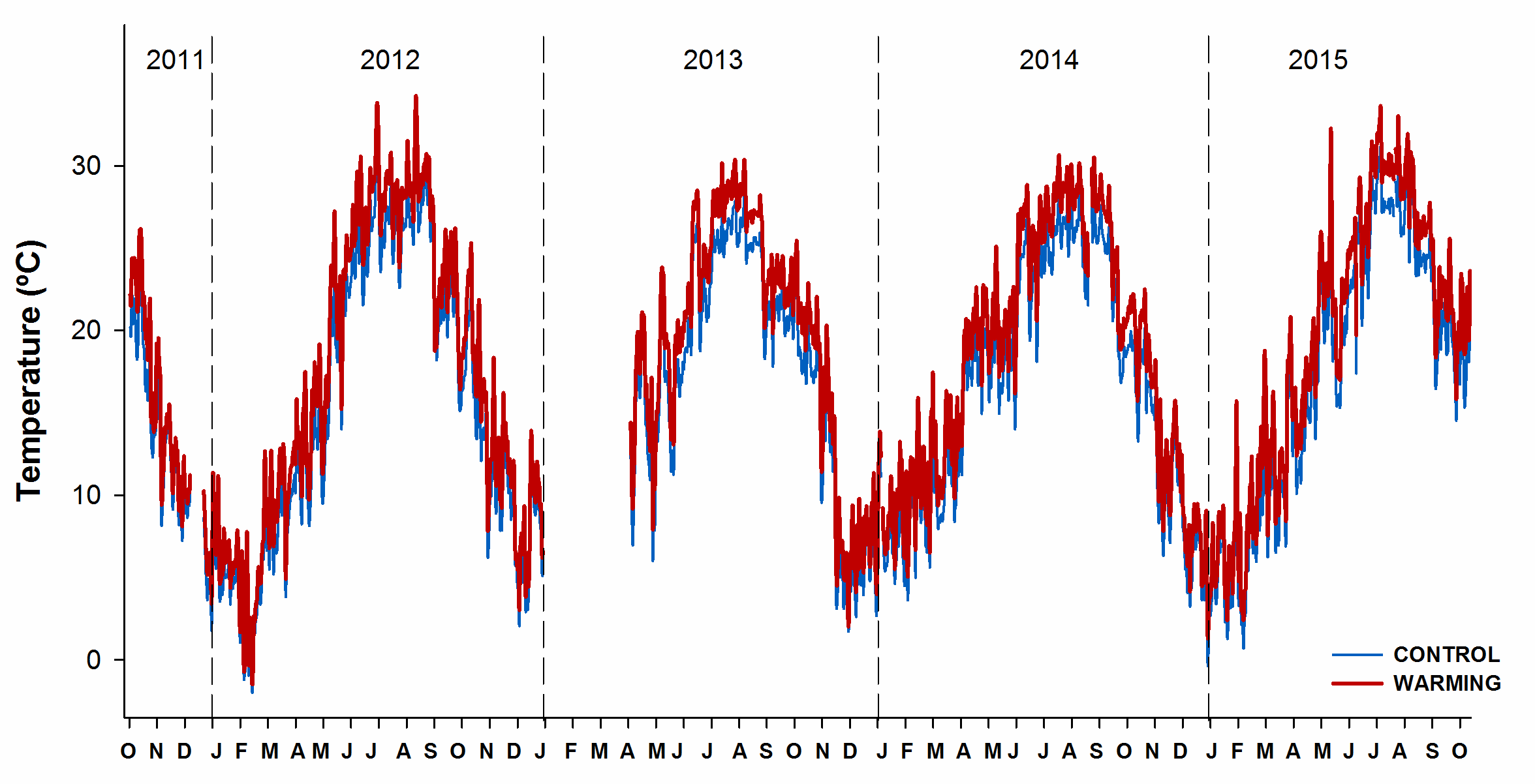


**Fig. S3** Rainfall distribution (blue bars) and soil water content (0-5 cm depth) measured by automated sensors on warmed and control plots throughout the 4-yr study period.


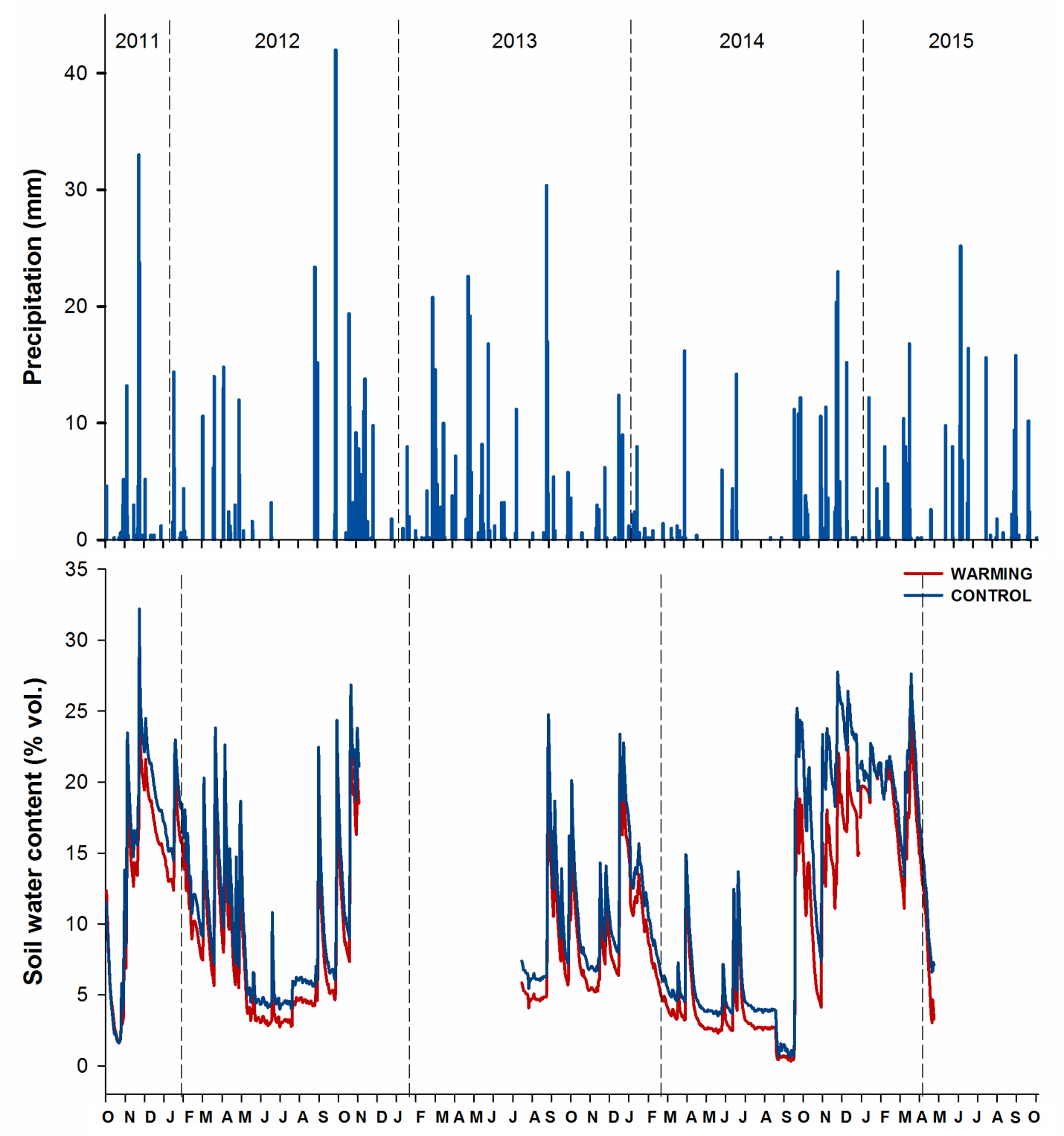

**Fig. S4.** Maximum efficiency of photosystem II under light conditions (Fv’:Fm’) in control and warmed plants at 10 different measurement dates spanning three consecutive growing seasons (data not available for 2015). Data represent means ± SE (n = 10-15).

**Table S1** Mean net photosynthetic rate (A), stomatal conductance (g_s_) and intrinsic water use efficiency (WUE_i_) at 11 different measurement dates spanning 4 growing seasons in warmed plants of OTCs installed in 2008 vs. warmed plants of OTCs installed in 2011. Mean values across measurements dates ± standard errors are shown (in each measurement date, N=7 for OTCs installed in 2011; N=5 for OTCs installed in 2008). The P values of the repeated measures ANOVA (using “year when OTCs were installed” as between-subject factor, and “study year” as within-subject factor) are also shown, indicating that neither leaf gas exchange parameter was significantly different between OTCs installed in 2008 and OTCs installed in 2011.

|  | **A**  **(μmol CO_2_ m^-2^ s^-1^)** | **g_s_**  **(mol H_2_O m^-2^ s^-1^)** | **WUE_i_**  **(μmol CO_2_/ mol H_2_O )** |
| --- | --- | --- | --- |
| **OTC (2008)** | 13.168 ± 0.796 | 0.285 ± 0.035 | 55.898 ± 3.700 |
| **OTC (2011)** | 13.933 ± 0.941 | 0.250 ± 0.041 | 59.576 ± 4.378 |
| **P** | 0.549 | 0.537 | 0.535 |

**Table S2**. Results of the repeated measures analysis of variance (RM-ANOVA) of net photosynthetic rate (A), stomatal conductance (g_s_), transpiration rate (E), intrinsic water use efficiency (WUE_i_), instantaneous water use efficiency (WUE_Inst_), maximum efficiency of photosystem II under light (Fv’:Fm’), and the actual photochemical efficiency of photosystem II (∅PSII). In these analyses, Warming and Time were the between-subject and within-subject factors, respectively. P values below 0.05 are highlighted in bold. All leaf gas exchange parameters were measured on 11 dates spanning 4 consecutive growing seasons (except for Fv’:Fm’ and ØPSII, which were not measured in April 2015).

|  |  | **A** | **g_s_** | **E** | **WUE_i_** | **WUE_inst_** | **Fv’:Fm’** | **ØPSII** |
| --- | --- | --- | --- | --- | --- | --- | --- | --- |
|  |  |  |  |  |  |  |  |  |
| **Warming** | **F** | 75.207 | 0.740 | 0.164 | 91.820 | 59.529 | 0.028 | 4.072 |
|  | **df** | 1,19 | 1,19 | 1,19 | 1,19 | 1,19 | 1.19 | 1,19 |
|  | **P** | **< 0.001** | 0.400 | 0.690 | **< 0.001** | **< 0.001** | 0.870 | 0.058 |
|  |  |  |  |  |  |  |  |  |
| **Time** | **F** | 31.157 | 14.851 | 13.365 | 10.367 | 15.538 | 6.253 | 7.212 |
|  | **df** | 10,190 | 10,190 | 10,190 | 10,190 | 10,190 | 9.171 | 9,171 |
|  | **P** | **< 0.001** | **< 0.001** | **< 0.001** | **< 0.001** | **< 0.001** | **< 0.001** | **< 0.001** |
|  |  |  |  |  |  |  |  |  |
| **Warming*Time** | **F** | 2.026 | 0.619 | 0.611 | 6.569 | 3.891 | 1.361 | 1.048 |
|  | **df** | 10,190 | 10,190 | 10,190 | 10,190 | 10,190 | 9.171 | 9,171 |
|  | **P** | **0.033** | 0.797 | 0.524 | **< 0.001** | **< 0.001** | 0.210 | 0.404 |

**Table S3**. Results of the repeated measures analysis of variance (RM-ANOVA) of leaf N and P concentrations, leaf mass, area and mass per unit area (LMA, data not available for April 2012), leaf isotopic composition (δ^13^C, δ^18^O, measured in April 2012, 2013, 2014, 2015), and post-summer plant survival (measured in October 2012, 2013, 2014 and 2015). Rest of legend as in Table S1.

|  |  | **Leaf N** | **Leaf P** | **Leaf mass** | **Leaf area** | **LMA** | **δ^13^C** | **δ^18^O** | **Plant survival** |
| --- | --- | --- | --- | --- | --- | --- | --- | --- | --- |
|  |  |  |  |  |  |  |  |  |  |
| **Warming** | **F** | 5.908 | 9.876 | 4.394 | 3.009 | 2.200 | 7.061 | 0.118 | 0.047 |
|  | **df** | 1.21 | 1.23 | 1.31 | 1.31 | 1.32 | 1.18 | 1.18 | 1.17 |
|  | **P** | **0.024** | **0.005** | **0.044** | 0.093 | 0.148 | **0.016** | 0.735 | 0.832 |
|  |  |  |  |  |  |  |  |  |  |
| **Time** | **F** | 66.511 | 19.211 | 14.834 | 63.970 | 100.311 | 37.368 | 23.813 | 10.719 |
|  | **df** | 3.63 | 3.69 | 2.62 | 2.62 | 2.64 | 3.54 | 3.54 | 3.51 |
|  | **P** | **< 0.001** | **< 0.001** | **< 0.001** | **< 0.001** | **< 0.001** | **< 0.001** | **< 0.001** | **< 0.001** |
|  |  |  |  |  |  |  |  |  |  |
| **Warming*Time** | **F** | 2.475 | 1.186 | 0.099 | 0.629 | 2.320 | 1.317 | 2.890 | 0.107 |
|  | **df** | 3.63 | 3.69 | 2.62 | 2.62 | 2.64 | 3.54 | 3.54 | 3.51 |
|  | **P** | 0.070 | 0.322 | 0.906 | 0.537 | 0.107 | 0.278 | **0.044** | 0.956 |
